# Recent Progress Regarding Materials and Structures of Triboelectric Nanogenerators for AR and VR

**DOI:** 10.3390/nano12081385

**Published:** 2022-04-18

**Authors:** Jinhao Si, Ruiguang Duan, Menglin Zhang, Xiaomin Liu

**Affiliations:** School of Physics and Microelectronics, Zhengzhou University, Zhengzhou 450001, China; jinhao_si@163.com (J.S.); ruiguang163@163.com (R.D.); menlinza@126.com (M.Z.)

**Keywords:** nanomaterials, triboelectric nanogenerator, AR and VR, self-powered sensing

## Abstract

With the continuous advancement in technology, electronic products used in augmented reality (AR) and virtual reality (VR) have gradually entered the public eye. As a result, the power supplies of these electronic devices have attracted more attention from scientists. Compared to traditional power sources, triboelectric nanogenerators (TENGs) are gradually being used for energy harvesting in self-powered sensing technology such as wearable flexible electronics, including AR and VR devices due to their small size, high conversion efficiency, and low energy consumption. As a result, TENGs are the most popular power supplies for AR and VR products. This article first summarizes the working mode and basic theory of TENGs, then reviews the TENG modules used in AR and VR devices, and finally summarizes the material selection and design methods used for TENG preparation. The friction layer of the TENG can be made of a variety of materials such as polymers, metals, and inorganic materials, and among these, polytetrafluoroethylene (PTFE) and polydimethylsiloxane (PDMS) are the most popular materials. To improve TENG performance, the friction layer material must be suitable. Therefore, for different application scenarios, the design methods of the TENG play an important role in its performance, and a reasonable selection of preparation materials and design methods can greatly improve the work efficiency of the TENG. Lastly, we summarize the current research status of nanogenerators, analyze and suggest future application fields, and summarize the main points of material selection.

## 1. Introduction

The triboelectricity phenomenon has a history that dates back more than 2000 years. When two objects rub against each other, one object loses electrons, while the other object gains electrons [1]. The basic working principle of a triboelectric nanogenerator (TENG) is to generate charges on a material surface by bringing two different friction materials into contact with each other to generate relative motion. When two different material layers produce relative motion, the two materials have different electron binding abilities, where one material inevitably loses electrons and the other material gains electrons. Therefore, the same number of charges with opposite polarities can be generated on the two material surfaces. According to the different electron binding abilities of the different materials, these materials can be arranged in order from high to low, known as the series of triboelectric materials, which can be used as a reference for material selection [2,3]. Due to the wide variety of triboelectric materials, a suitable pair of triboelectric materials depends not only on their chemical composition but also on their physical properties, such as hardness, toughness, and shape [4]. Therefore, selecting an appropriate pair of triboelectric materials is difficult, and it was not until the advent of the first triboelectric nanogenerator in 2012 that this problem was effectively solved [5]. Currently, the most popular materials include polytetrafluoroethylene (PTFE), polydimethylsiloxane (PDMS), and fluorinated ethylene propylene (FEP). Researchers have conducted applicability studies on various materials to explore their performance characteristics for different application scenarios, and their TENG applications include tidal energy harvesting [5,6,7,8,9], self-powered sensors [10,11,12], and wearable flexible electronic devices [13,14,15,16,17,18]. PTFE is often used to manufacture TENG modules in wearable flexible electronic devices, as PTFE film is non-toxic and has the characteristics of flexibility, transparency, and mechanical stability [19,20,21]. In addition, PTFE film corona offers a fast charging time with high efficiency, based on the PTFE film preparation of TENG. It also has a generally high energy conversion efficiency, high output voltage, and stable characteristics [22]. PDMS is often used in self-powered, sensing, and flexible electronic devices because of its inherent elasticity and excellent biocompatibility [23,24,25,26]. In addition, PDMS films can achieve full contact with skin as they can freely distort and deform, resulting in widespread interest in the material industry [27,28]. When PDMS is embedded with microstructures and various sensitive materials on the surface, the sensor exhibits high sensitivity, good linearity, and strong flexibility. More importantly, the microstructure of the PDMS film can effectively reduce adhesion between materials, thus promoting relative sliding between the friction layers. Therefore, there is an urgent need to use PDMS elastomers as the main body to improve the friction characteristics of the friction layer, and to create TENG with combined excellent flexibility and high output performance [29].

**Figure 1:**
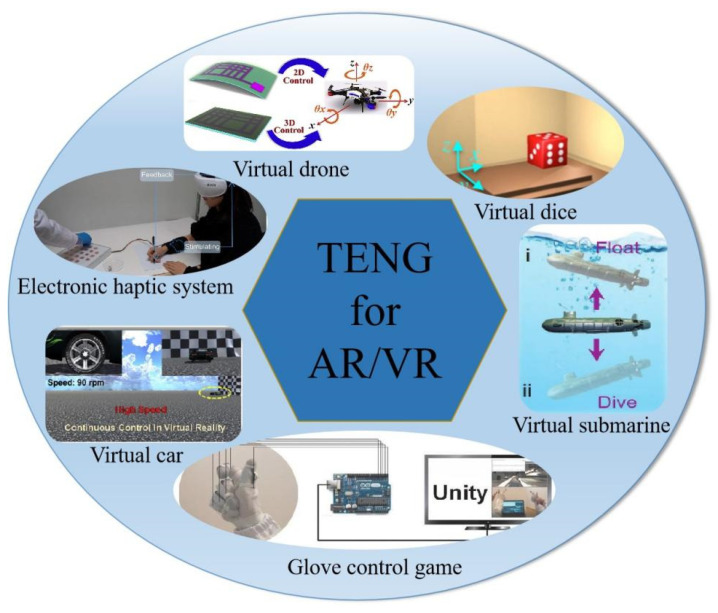
Application of TENG in AR and VR. Reprinted with permission from ref. [39]. Copyright 2021 Science Advances; Reprinted with permission from ref. [40]. Copyright 2019 Nano Energy; Reprinted with permission from ref. [41]. Copyright 2018 Nano Energy; Reprinted with permission from ref. [34]. Copyright 2021 Advanced Materials Technologies; Reprinted with permission from ref. [42]. Copyright 2020 Advanced Science; Reprinted with permission from ref. [43]. Copyright 2020 Nano Energy.

TENG uses the principle of triboelectricity, where the friction between two materials is used to convert mechanical energy into other forms. Compared to traditional power supplies, TENGs can convert scattered and difficult-to-use mechanical energy into electrical energy [30]. Furthermore, these materials have advantages such as low cost, a simple design, convenient carrying, a high conversion rate, and a variety of material choices [31,32]. Due to the rapid development of artificial intelligence, there is an increasing demand for green energy and wearable electronic products. Compared to other forms of green energy, mechanical energy is the most widely distributed energy in the natural environment and daily life, and it is not affected by the external environment [33]. Therefore, determining how to use TENGs to convert mechanical energy into electrical energy and maximize conversion efficiency has received considerable research attention. Moreover, as a result of the rapid development of the Internet of Things industry, human–computer interactions and intelligent perception have gradually entered our daily lives, and human–computer interactions are no longer limited to voice commands [34]. For example, gesture recognition can be used for target control, and gesture recognition has been used in VR and AR with good developmental prospects. Among them, TENG-based AR and VR technology is on the rise (Figure 1). Compared to traditional control methods, some AR and VR applications have allowed users to experience convenient and intelligent operations [35] such as VR glasses [36,37], VR gloves [15,38] and flexible patches. VR gloves can replace the traditional computer mouse and keyboard accessories to achieve a non-contact operating computer interface. Furthermore, through intelligent perception technology, game players can have a more immersive experience.

This article discussed the selection of TENG materials including PTFE, PDMS, and other materials, and analyzed the advantages and disadvantages of the different materials. Subsequently, TENG material structures and designs for AR and VR equipment were introduced, and the applications of AR and VR equipment based on TENG were summarized, including gloves and flexible patches. Furthermore, TENG was used as a self-powered sensor module to realize human–computer interactions between the human and the computer screen, realize the intelligent perception of virtual objects, and achieve control. Finally, this article summarized the developmental status of TENG worldwide and discussed the developmental prospects and challenges of further TENG applications for AR and VR in the future.

## 2. The Basic Theory for TENG

**Figure 2:**
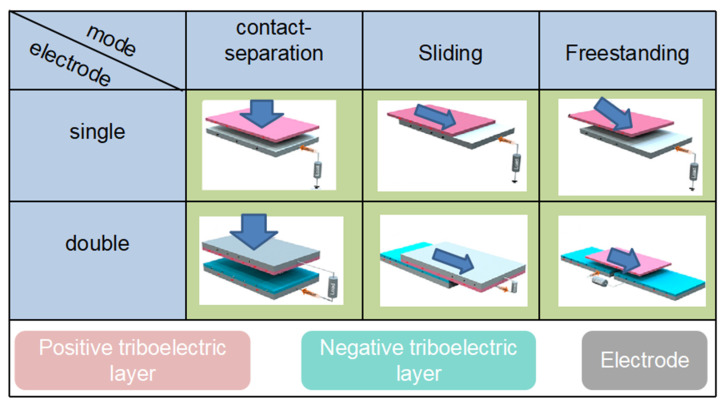
Working modes of TENG.

The working modes of TENG can be generally divided into three categories: contact-separation mode, sliding mode, and freestanding mode. Each mode can be further divided into single and double electrodes [44], as shown in Figure 2. The contact-separation mode is composed of two types of dielectrics that face each other, which can be divided into positive and negative electrodes. When the two electrodes are in contact, the dielectric surface generates a charge [45]. However, when the two electrodes are separated, a potential difference is generated on the surface of the two dielectrics, and the charge from the positive electrode is transferred to the negative electrode. When the two electrodes come into contact again, the electrons move in opposite directions, and an alternating current is generated through this alternating back-and-forth process [46]. The sliding mode consists of the relative motion of two dielectrics, where the surfaces of the two materials generate charges due to friction; thus, a potential difference forms between the positive and negative electrodes [47,48,49]. The change in the effective contact area of the two dielectrics during the relative motion causes a potential difference to occur, while periodic changes result in alternating current [50]. Freestanding is based on the natural friction between the friction material and the surrounding air. In this mode, the charge on the triboelectric layer can last a long time; thus, no external drive is required [51,52]. Moreover, the movement of the triboelectric layers is irregular and an asymmetric electric field is formed, generating triboelectric energy between the triboelectric layers. Compared to the first two modes, no direct contact occurs between the freestanding triboelectric layers; thus, wear between materials is reduced. In the case of the single-electrode configuration, one output terminal is connected to the electrode, and the other output terminal is virtually grounded, and in the double-electrode configuration, both output terminals are connected to the electrode. Among these configurations, the advantages of dual electrodes include higher flexibility and a wider range of motion [53]. For example, when a car is running, the tire and the ground is triboelectrically charged. The tire can be used as an output electrode while the ground acts as another electrode. In this case, a single-electrode mode would be suitable. However, because of the lack of a real reference electrode, the resulting voltage and current may be unstable, while the double-electrode modes do not have such a problem [54,55]. Therefore, choosing the correct electrode configuration for each application is important.

**Figure 3:**
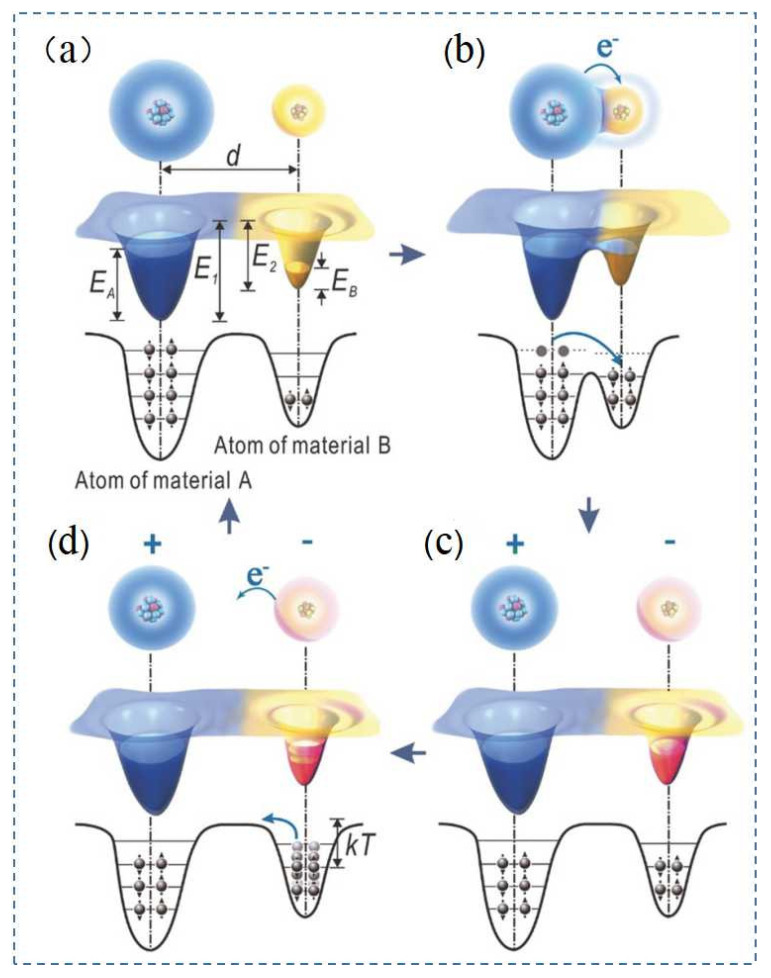
Electron transfer model of TENG. Reprinted with permission from ref. [56]. Copyright 2018 Advanced Materials. (**a**) Material A and material B before contact; (**b**) the two materials in contact; (**c**) the two materials separated; and (**d**) with increasing temperature, electric charges were released by the atoms.

The charge transfer mechanism in triboelectric electrification has always been a vexing conundrum among scientists. In 2017, Zhonglin Wang et al. proposed a new method to investigate the charge transfer changes of TENGs with temperature [56], and determined why the charges generated in triboelectric electrification were easily retained in the material at room temperature. For the polymers and amorphous materials summarized in this paper, an electron cloud-potential well model proposed by Zhonglin Wang et al. was used to explain the charge transfer mechanism in most polymer materials [57]. As shown in Figure 3, electron clouds were formed by the electrons, which were localized within the atoms and occupied specific atomic orbitals. The atoms were represented by potential wells, whose outer layers were bound by the electrons that formed the atomic electron clouds [58]. As shown in Figure 3a,d the distance between the electron clouds, EA, and EB, consisted of the occupied energy levels of the electrons in the atoms of materials A and B, E1 and E2 were the potential energies required for the electrons to escape from the material surface, and EA and EB were smaller than E1 and E2, respectively. Before the two materials came into contact, electrons could not transfer due to the trapping effect of the potential wells. When material A made contact with material B, the single potential well became a double well potential, and the electrons could move from material A to material B [59], as shown in Figure 3b. When materials A and B were separated, most of the electrons that were transferred to material B were retained due to the potential energy E2 in material B [60], as shown in Figure 3c. Therefore, positively charged material A and the negatively charged material B exhibited a contact electrification effect. As the temperature increased, the electrons contained more energy, which made it easier to jump out of the potential well and return to the original material, as shown in Figure 3d. This model elucidated why the charge generated by contact electrification remained constant due to the potential barrier of the material.

What is worth mentioning is that Wang Zhonglin expanded the most famous Maxwell equations in electrodynamics in 2021 to develop the theoretical framework of nanogenerators [61]. The expanded Maxwell equations include not only all the connotations of the classical Maxwell equations but also the electromagnetic coupling effect resulting from the motion of the charged medium, and the theoretical architecture of the nanogenerator. So far, worldwide attention has been paid to the research of nanogenerators because of their important applications in micro-nano energy, self-driven sensing, blue energy, and artificial intelligence [62,63]. Nanogenerators convert mechanical energy into electrical signals with displacement current as a driving force. In 2017, Wang Zhonglin expanded the expression of displacement current for the first time to derive the output power of nanogenerators [5]. In 2019, the analytically deduced transport equation of nanogenerators and the analytical solutions to the four modes of TENG given by Wang Zhonglin laid the overall theoretical framework of nanogenerators and formed the theoretical basis for the development of this discipline [61,64].

## 3. Material Selection for TENG

There are many types of factors that affect TENG performance, and among them, material selection plays a decisive role. This is because the physical and chemical properties of triboelectric materials can directly change TENG performance [1,4]. Several parameters such as power density stability, flexibility, and sustainability must be considered when designing TENG for specific applications [3]. In addition to high frictional electrical properties, there are different requirements for materials depending on the application. Some materials are suitable for energy collection, other materials are suitable for supercapacitors in self-powered sensing systems, while two-dimensional materials have bi-functional properties [1,44]. For example, Vimal Kumar Mariappana et al. discovered a paper-like carbyne material [65], and TENG prepared with this thin film exhibited good power density and energy density. In addition, due to its extensibility and stability, the two-dimensional material could be used to develop self-powered implantable nanodevices repairable by the human body [66]. Minsu Seol et al. studied the triboelectric charge behavior of various two-dimensional materials such as MoS2, WS2, and graphene oxide in the triboelectric series, and determined that the charge transfer efficiency between the tribomaterials had an obvious relationship with the effective work function [31]. In addition, the charge characteristics of the friction material could be modified by chemical doping. Hypothetically, to generate more charges or obtain higher output from TENG, two materials with significantly different charge affinities are preferred. Theoretically, the greater the difference in charge affinity of the two materials, the stronger the output voltage and current of the TENG prepared from the two materials. However, in practice, the two materials with the largest difference in charge affinity are not selected, as triboelectric electrification between the two materials depend not only on their chemical composition but also on other physical properties such as elasticity, friction, and surface topography [67]. The triboelectric effect of the materials is usually represented by the surface charge density of the material. Even for the same material, different triboelectric charging processes result in different surface charge densities. By analyzing the above nanomaterials, studies have shown that many have good triboelectric properties; moreover, the nanomaterials also exhibit good piezoelectric properties. One such example is polylactic acid (PLA). As a biomaterial, PLA has been widely used due to its unique advantages such as biocompatibility, biodegradability, and piezoelectricity [68,69]. The piezoelectric properties of PLA are related to its crystallinity, crystal phase, and temperature, and the piezoelectric constant of PLA is a function of its crystallinity [70,71,72,73]. Thus, by determining the piezoelectric constant of nanomaterials, one can compare the piezoelectric or triboelectric properties of materials [74,75,76].

**Figure 4:**
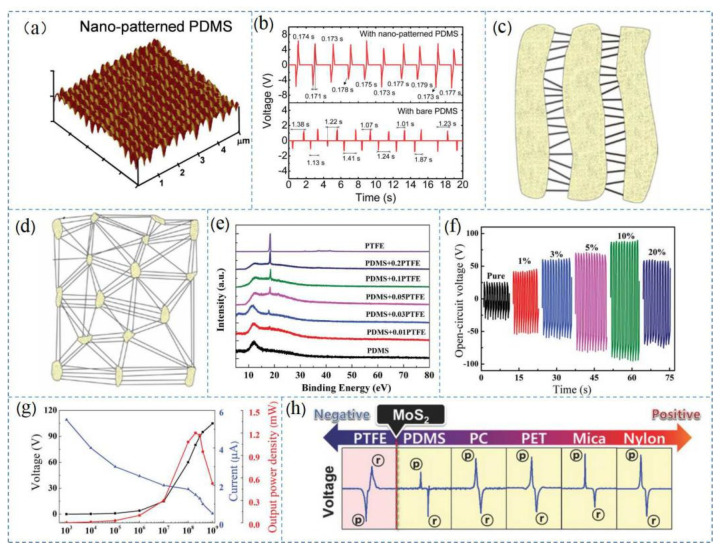
Preparation and characterization of the materials. (**a**) AFM images of PDMS; (**b**) output voltages of the triboelectric nanogenerators based on PDMS. Reprinted with permission from ref. [95]. Copyright 2014 Royal Society of Chemistry; (**c**,**d**) PTFE structures during preparation. Reprinted with permission from ref. [96]. Copyright 2018 Journal of Membrane Science; (**e**) XRD patterns of the PDMS-PTFE films; (**f**) output voltages of the PDMS-PTFE films; (**g**) relationship between output performance of the PDMS-PTFE films and the external load. Reprinted with permission from ref. [81]. Copyright 2019, Advanced Electronic Materials; and (**h**) output voltage of the triboelectric series of materials. Reprinted with permission from ref. [31]. Copyright 2018 Advanced Materials.

After reviewing numerous TENG-related articles, we concluded that the following materials were most widely used: PTFE [19,22,77,78,79,80,81], PDMS [29,82,83,84,85], FEP [86,87,88], polyester polyethylene terephthalate (PET) [30,89], and graphene [61,85,86]. Figure 4 presents the preparation and characterization of triboelectric materials such as PTFE and PDMS. The nanostructure on the surface of the PDMS material is clearly visible in Figure 4a, and the TENG prepared from this thin film exhibited high transparency and stable voltage output characteristics, as shown in Figure 4b. Figure 4c,d shows the structure diagrams at different stages during PTFE preparation according to the biaxial stretching method, and finally, a porous PTFE film was prepared. Figure 4e–g shows characterization of the mixed PTFE and PDMS films [81]. The voltage and current variation characteristics of the PDMS films with different PTFE content were analyzed, indicating that the triboelectric properties of the PDMS films could be improved by adding a suitable amount of PTFE to the PDMS films. Figure 4h shows a comparison of the charge affinity among the triboelectric material family, showing that MOS2 was between PTFE and PDMS. This order also indicated ordering of the triboelectric properties among the individual materials. These materials were also used in the AR and VR products reviewed in this paper. In addition to the above triboelectric materials, cellulosic materials [90] have attracted significant attention due to their high reproducibility and production efficiency [32], and the output power densities of cellulose-based TENG have greatly improved [91]. Currently, altering the chemical properties of engineered polymers is of interest, as the modification method can regulate the ability of the material surfaces to rub against each other and generate electric charges, thereby increasing the power output of the TENG [1]. For example, studies have shown that the hydrophobicity and electrical conductivity of the TENG materials could be changed by a coating process. Furthermore, inorganic triboelectric two-dimensional materials such as MoS2 [92] and WS [93,94] have received attention, and the charge mechanisms of these materials are well known. However, these materials are difficult to prepare due to their physical properties such as large-area single crystal films. Therefore, research on these materials is still limited.

Currently, there are four basic types of triboelectric nanogenerators: single electrode [97], contact separation [98], lateral sliding [99], and independent triboelectric layer [60] modes. Triboelectric materials are placed in vertical contact in the single electrode and contact separation modes, and there are many materials to choose from. Unlike the first two modes, lateral sliding and independent triboelectric layer modes have specific requirements for materials, namely low friction coefficients. If the friction coefficient of the material is high, the relative sliding between the two materials wear away the material. Thus, PTFE and PDMS have been widely used in these two modes due to their low friction coefficients.

## 4. The Structures of TENG

**Figure 5:**
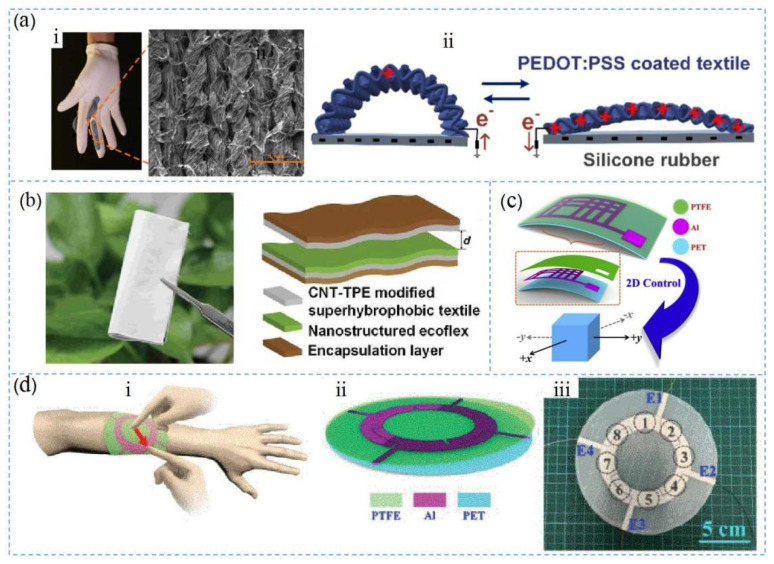
Materials of the wearable TENG: (**a**) material structures of the textiles. Reprinted with permission from ref. [38]. Copyright 2019 Nano Energy; (**b**) material structure of the superhydrophobic textile TENG. Reprinted with permission from ref. [42]. Copyright 2020 Advanced Science; (**c**) material structure of TENG to control a virtual UAV, Reprinted with permission from ref. [40]. Copyright 2019 Nano Energy; (**d**) material structure of triboelectric patch on a human arm. Reprinted with permission from ref. [104]. Copyright 2020 IEEE.

Over the past two years, nanogenerators have been used more frequently in wearable devices, especially gloves. Figure 5a shows two TENG configurations proposed by Li et al. to meet the requirements for full human-machine interface (HMI) functionality and simplified signal processing capabilities. The TENG was composed of PEDOT (poly(3,4-ethylenedioxythiophene)), which consisted of a PSS (poly(styrene sulfonate)) coating with silicone [100]. PEDOT:PSS was chosen because the material offers many excellent properties such as its physical and chemical properties, which are stable at normal temperature and pressure, and the material exhibits good transparency, good electrical conductivity, easy preparation, strong film-forming abilities, and a low cost. As shown in Figure 5b, Feng et al. proposed a simple coating method for carbon nanotubes (CNTs) [101], achieving super-hydrophobic textiles with improved output performance [102]. The mechanism of the material exhibiting superhydrophobicity while also maintaining excellent triboelectric properties was due to two factors: First, water droplets had difficulty adhering to the surfaces of CNT and TPE materials due to the obstruction of the superhydrophobic interface, which improved its waterproof performance. Second, because the rough structure of the CNT and TPE composite surface increased the actual surface area, the friction force was enhanced and the triboelectric effect became more pronounced. In addition, superhydrophobic textiles can quickly resolve moisture and dry quickly in wet environments. Using this textile solved the problem of the output voltage being susceptible to humidity. As shown in Figure 5c, a flexible single-electrode two-dimensional control patch was produced, which could realize in-plane two-dimensional motion control. The control patch consisted of only three thin layers, namely a PET base layer, a grasping pattern Al electrode layer, and a PTFE friction layer [103]. The working principle of the two-dimensional control patch was based on contact electrification and electrostatic induction between the PTFE and the glove. Due to the different binding abilities to electrons of the two materials, a potential difference was generated between the PTFE friction surface and the glove. Thus, voltage output was generated on the peripheral circuit, which acted as a power source. Figure 5d shows the triboelectric interaction patch with four sensing electrodes, as proposed by Qiongfeng et al. As a flexible multifunctional human-machine interface, it was used to detect various human–machine interactions [66]. This patch was also composed of a PET substrate, an open-loop aluminum electrode (E1~E4), and a PTFE friction layer.

**Figure 6:**
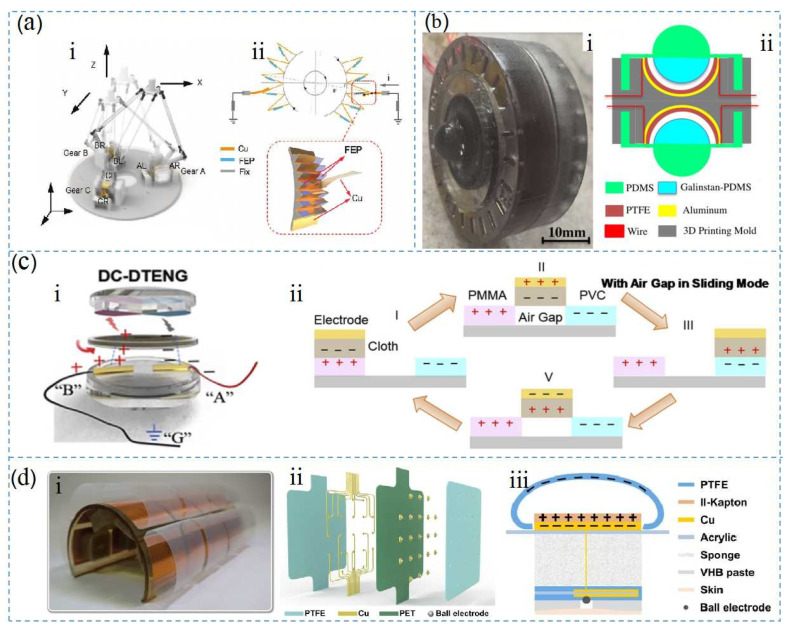
Structure of the VR products: (**a**) TENG fabricated from the FEP film used for mobile platform control, Reprinted with permission from ref. [34]. Copyright 2021 Advanced Materials Technologies; (**b**) material structures and physical photos of a self-powered sensor made with PDMS and PTFE, Reprinted with permission from ref. [41]. Copyright 2018 Nano Energy; (**c**) internal structure diagram and material hierarchy diagram of DC-DTENG, Reprinted with permission from ref. [43]. Copyright 2020 Nano Energy; (**d**) material structure of an electro-tactile system based on TENG and spherical electrode array formation, Reprinted with permission from ref. [39]. Copyright 2021 Science Advances.

Figure 6a shows a self-powered delta-parallel human–machine interface (DT-HMI), which was proposed by Cheng et al. In the DT-HMI, the friction materials used by the TENG were copper sheets and FEP films. During the initial state, the surface charge of the copper sheet was balanced. When the gear rotated clockwise, the positive charges in the FEP film and copper sheet were offset, causing the negative charges on the copper sheet to repel the ground [105]. When the current flowed from the ground to the copper electrode, the copper electrode generated a negative pulse. However, detaching the copper sheet and the FEP membrane produced a positive current signal. Figure 6b shows a TENG-based three-dimensional control sensor proposed by Tao et al., which was used to detect and control the movement of objects in a three-dimensional (3D) virtual space. This device consisted of two identical non-planar TENG sensing modules [106], and the module was composed of two hemispheres and PTFE film. The PTFE structure was designed with a hemispherical bottom in order to increase the contact area of the friction material, thereby increasing the triboelectric charge density of the TENG. In addition, Zhu et al. developed a continuous DC nanogenerator using one-way charge transport and double-cross TENG, as shown in Figure 6c [107]. The rotating disc was composed of porous cloth and the intermediate material cloth was assembled with an acrylic ring [108,109], while a dielectric pair was connected to the top stator. The dielectric materials consisted of PMMA (poly(methyl methacrylate)) and PVC (polyvinyl chloride), respectively, where the PMMA dielectric exhibited a positive surface charge upon contact with the cloth, and the PVC dielectric exhibited a negative surface charge on the cloth during operation [110]. Many materials can be used as intermediate materials when arranged in the order of the three frictional polar materials, such as natural rubber and paper. Considering PVC and PMMA, Zhu et al. found that a porous cloth with friction-induced polarity reversal was an ideal intermediate material due to its mechanical strength and flexibility as a friction layer [43]. The experimental results showed that the larger the contact area between the intermediate material and the medium, the better the triboelectric performance, the higher the voltage obtained, and the more charges that were transferred. Therefore, the contact area between the intermediate material and the dielectric material should be increased as much as possible. The PMMA film was located on the left side on one quarter of the disk, and PVC was located on the right side on the other quarter of the disk. Bottom electrodes A and B consisted of wire electrodes placed in the bottom stator, and the wire electrodes were made of cloth coated with nickel metal. This electrode was chosen due to its mechanical properties, as it was flexible and easily integrated into the equipment [111]. Figure 6d shows an electronic system (ET) based on a TENG for a virtual haptic experience, where the different polymer films were made of PTFE, Kapton, PET, and PEN [112]. Regarding the selection of materials for the ET interface, PTFE was selected as a negative triboelectric material because of its good charging performance and low cost after corona polarization. Ion bombardment technology has recently been used to modify capacitor energy storage materials, and experiments showed that this method significantly improved the triboelectric properties of Kapton film [39]. In this study, a similar ion bombardment technology was used to improve and enhance TENG in the ET system. In addition, as shown in our previous work, ion radiation could not be improved due to the weak thermal stability of the PET film [39]. Therefore, in this study, because polyethylene naphthalate (PEN) had high thermal stability, we selected it as an alternative material.

## 5. Applications of TENG in AR and VR

**Figure 7:**
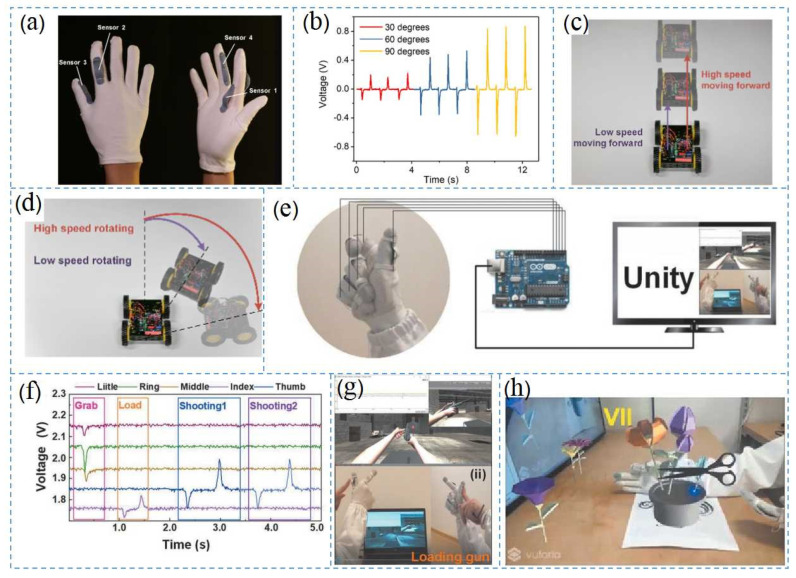
Applications of TENG-based VR gloves: (**a**) self-powered gloves using a PEDOT:PSS coating process; (**b**) voltage output of the sensor when the index finger was bent at different angles; (**c**,**d**) different driving states of wireless vehicles controlled by gloves. Reprinted with permission from ref. [38]. Copyright 2019 Nano Energy; (**e**) schematic diagram of machine learning glove-controlled gunplay based on self-powered conductive superhydrophobic triboelectric textiles; (**f**) signal modes for fetching, loading, and firing; (**g**) screenshot of gun loading action in Unity VR space; and (**h**) virtual illustration of glove control. Reprinted with permission from ref. [42]. Copyright 2020 Advanced Science.

Qiongfeng et al. proposed a TENG based on two configurations to realize more HMI functions and make signal processing easier. Each sensor consisted of a PEDOT:PSS-coated textile tape and the gloves were coated with a layer of silicone film (Figure 7a) [113]. This type of HMI was used for unmanned driving technology, and the operation technology was simple and intuitive, as the four sensors were represented by different finger movements. Figure 7b shows the real-time outputs of the sensors when the fingers were bent downward at different angles at the same speed [114]. This HMI could control the car, drive it in different directions, and the running state of the car could be changed according to the finger bending angle and strength of the operator (Figure 7c,d).

To address the issue of human sweat on glove performance, a simple CNT and TPE coating method was proposed by Qiongfeng et al., which is shown in Figure 7e, to achieve superhydrophobic triboelectric textiles with improved performance [115,116]. The super-hydrophobic fabric recovered quickly from high humidity conditions seven times faster than the original fabric, and the triboelectric properties were three times better. Superhydrophobic triboelectric textiles collected biomechanical energy from human activities at four times the power density of the original textiles [117]. In a high humidity environment, superhydrophobic textiles with anti-perspiration properties can monitor human movements without obvious output voltage deterioration. Figure 7g,h shows the applications of superhydrophobic triboelectric gloves in an AR space. Figure 7g shows the control of firearms in a 3D shooting game, where each sensor on the glove was connected to a single-chip microcomputer and controlled through a serial port. Python was used for real-time processing and analysis, and the commands were sent to Unity. The shooter controls included “grab the gun”, “reload”, and “shoot”, which were achieved through three different signal modes. First, the user bent their fingers so that the super-hydrophobic textile was in full contact with the ecoflex, and the characters in Unity responded to this command and grabbed the gun. In the second stage, the user pressed the sensor button to trigger the refill command, and the index finger was bent for the shooting command. The flower arrangements shown in Figure 7h were based on a variety of gestures such as “water”, “spin”, “light”, “pick”, “trim”, and “stop”. After training in deep learning model, the average accuracy for gesture recognition reached 96.75% [118].

**Figure 8:**
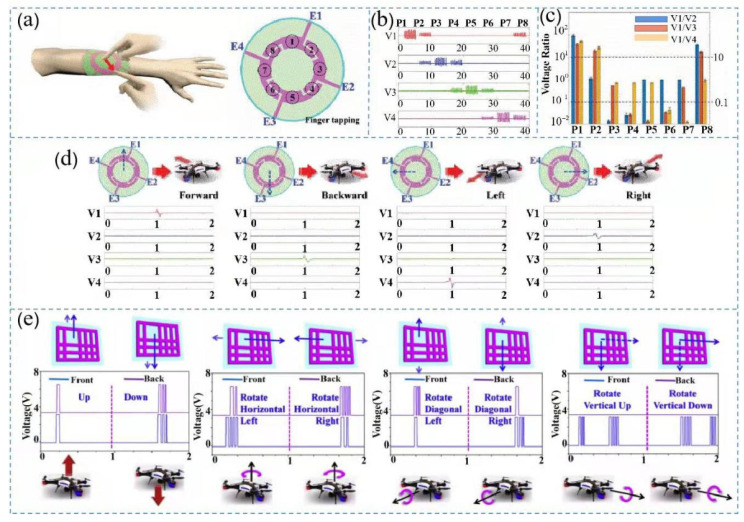
Virtual UAV control based on TENG: (**a**) triboelectric patch attached to the skin of a human arm; (**b**,**c**) output signals corresponding to finger tapping and sliding of the patch; (**d**) triboelectric patch controlling signal output corresponding to the horizontal movement of the UAV, Reprinted with permission from ref. [104]. Copyright 2020 IEEE; and (**e**) corresponding signal output of the UAV when it moved through 3D space. Reprinted with permission from ref. [40]. Copyright 2019 Nano Energy.

Zixuan et al. proposed a triboelectric interaction patch with only four sensing electrodes for a flexible multifunctional human–machine interface to detect various action signals, allowing the user to set an operating area in advance to control the input and output relationship of the four electrodes to achieve position detection (Figure 8a) [119]. The triboelectric patch exhibited accurate sensing ability and could adapt to finger tapping, sliding, and other common finger movements. The TENG was operated by sliding fingers on the eight defined points shown in Figure 8b,c. Using the ratio of each voltage, the position perception performance of these eight points in the two scenarios could be identified. Thus, the triboelectric patch could be used for UAV control, and the output signal and corresponding UAV movements are shown in Figure 8d [120].

To further improve the practicality of the equipment and achieve a minimalist control interface, Qiongfeng et al. developed a control patch with a single electrode to control a UAV in a virtual space and demonstrated the 3D control capabilities of the equipment (Figure 8e). The control system consisted of a 3D control patch, which was used to generate the dual-channel control signals [121], a processing circuit, and an MCU used to calculate the number of dual-channel output peak values. After receiving the control commands, the computer generated the corresponding actions for the UAV. Figure 8e shows the two-channel signals and six degrees of freedom obtained after the 3D control patch was processed by the circuit.

**Figure 9:**
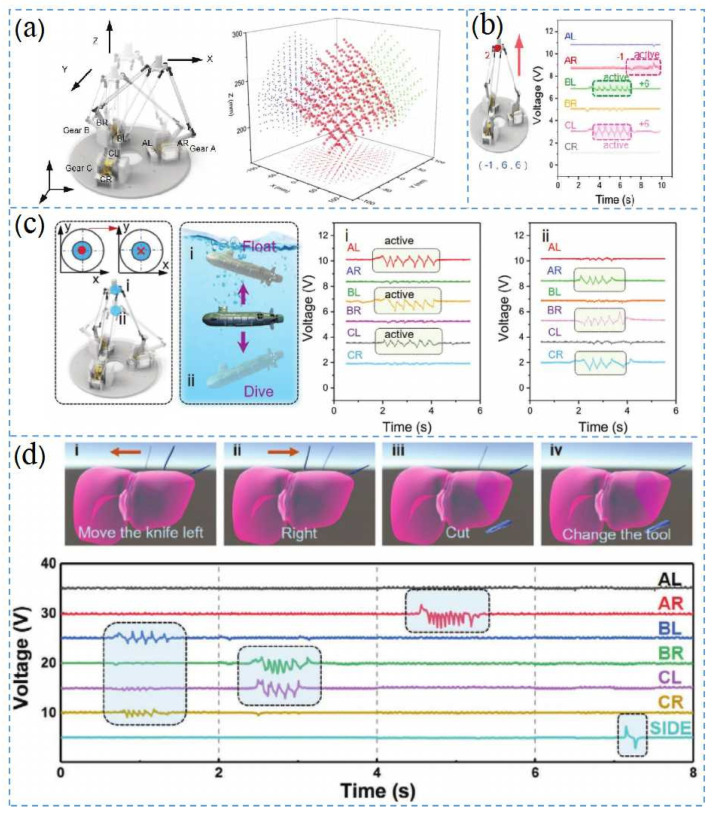
Application of delta-parallel-inspired human–machine interface (DT-HMI) on AR and VR, Reprinted with permission from ref. [34]. Copyright 2021 Advanced Materials Technologies. (**a**) delta manipulator movement controlling the platform based on self-powered triboelectric nanogenerator and its reachable spatial points; (**b**) output electrical signal of the platform when the manipulator moved; (**c**) platform controlling the submarine’s movement during rising and diving and corresponding output voltage changes; and (**d**) augmented reality surgery training program for liver resection with DT-HMI.

Figure 9a shows a new self-powered DT-HMI which was combined with the output signal from a TENG sensor, achieving 2/3D control for VR and AR interactions, robotics, and other applications. The DT-HMI consisted of three parallel branches connected between the movable platform and the fixed base, where each branch consisted of a drive bar and a passive bar. At the end of the drive lever, there were three sensitive gears (A, B, and C), each with two TENGs for clockwise and counterclockwise angle identification. Since each gear had two TENGs, there was a total of six TENGs for three gears. The electrical signals of the six TENGs produced different peaks in different operating modes (Figure 9b). Figure 9c shows an application that controlled the movement of a virtual submarine through a mobile platform. By identifying the digital status signals from the six TENG outputs, different operation commands of the submarine in different states such as diving, floating, forward and backward movement, and movement relative to the moving platform were defined. For 3D operation of the submarine model floating and diving, the mobile platform would move upward or downward, triggering the entire left side TENG sensing of the gears or the entire right side TENG sensing of the gears. Similarly, moving in different directions through gear commands with different control codes achieved different motion states of the submarines in the water. As shown in Figure 9d [122], DT-HMI was used for AR liver resection in virtual minimally invasive surgery, and the camera was used for a plane image recognition control script, which mainly consisted of a scalpel, pliers, liver resection, and instrument switching. The TENG sensor gear was mainly used for instrument operation, and the AR output signal represented the movement states. Although the procedure above demonstrated a simple operation, it still showed the value of the TENG for surgical applications.

**Figure 10:**
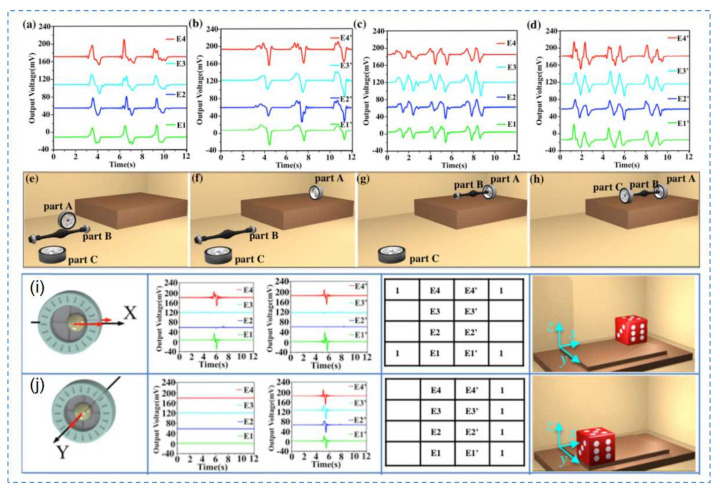
Using 3D self-powered sensor AR interface of virtual assembly, Reprinted with permission from ref. [41]. Copyright 2018 Nano Energy. (**a**–**h**) part assembly process and corresponding voltage changes; and (**i**,**j**) relationship between six-axis operation strategy for 3D control sensor and voltage curve with AR interface control dice.

Due to the large size, poor portability, and difficult operation of the DT-HMI, a new TENG-based sensor was developed to control the movement of a virtual object. The structure of the sensor was three-dimensional, symmetrical, and composed of eight independent sensing electrodes and two touch balls, with human–computer interaction functions, to realize three-dimensional force information perception and VR control [123]. Thus, by analyzing the relevant properties of the force and the electrode, the triboelectric mechanism was used for the first time to detect a six-axis direction in 3D space. As a result, the researchers successfully realized the control function of the sensor in a VR interface. To avoid electromagnetic interference between the eight sensing signals, the following method was adopted [124]. First, the circuit in the A/D converter used differential input. Then, the sensor was set with a high threshold trigger in the software to distinguish instructions from the interference. Figure 10a–h shows a schematic diagram of the controlling parts for virtual assembly and the corresponding axial voltage changes. By controlling the movements of each part and by selecting and releasing certain instructions, the three parts operated in turn to complete the assembly process [125]. Figure 10i,j shows a schematic depicting the controlled movements of virtual dice in a game.

**Figure 11:**
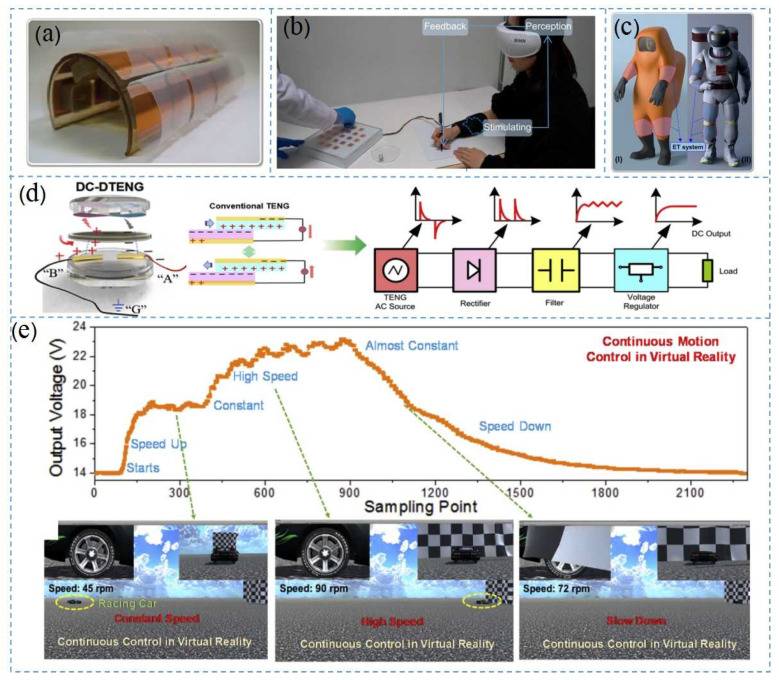
VR application of TENG: (**a**) unit structure of a self-powered, painless, and highly sensitive electro-tactile (ET) system based on TENG and spherical electrode arrays; (**b**) ET system applied to a dynamic braille display; (**c**) positive pressure suits and spacesuits equipped with ET systems, Reprinted with permission from ref. [39]. Copyright 2021 Science Advances; (**d**) DC-DTENG using an external electric device for DC output; and (**e**) DC-DTENG controlling the output voltage when the virtual vehicle performed at different speeds, Reprinted with permission from ref. [43]. Copyright 2020 Nano Energy.

Tactile sensing plays an important role in VR and AR systems. Based on the ET interface formed by the TENG and spherical electrode array, Yuxiang et al. proposed a self-powered, painless, and highly sensitive ET system for a virtual tactile experience [126]. The structure of the ET unit is shown in Figure 11a [127]. This TENG-based ET system could be used in many fields, including for virtual haptic displays, Braille commands (Figure 11b) [128], intelligent protective clothing (Figure 11c), and even neural stimulation. The conversion of mechanical energy into direct current was also important for the next generation of self-contained Internet of Things and real-time virtual reality control. By using a porous material with frictional polarity reversal as the charge transport carrier, the charge was transferred unidirectionally between the hyper-negative and hyper-positive materials and the repulsive discharge through the wire electrode [129], forming a stable DC output (Figure 11d). Due to the charge transfer and repulsive discharges, obtaining a much higher DC output voltage was easier compared to the air breakdown mechanism [130]. Figure 11e shows a racing control game in a virtual space, based on continuous DC double-crossing TENGs (DC-DTENG). The continuous control of acceleration, constant speed, and further acceleration and deceleration was identified with real-time connection in real and virtual space [131]. Notably, the car in the virtual space was completely controlled by the corresponding continuous output signals of the real DC-DTENG with mechanical activity.

In this paper, various TENG-based designs for AR and VR applications were summarized. Among them, flexible wearable devices made from two-dimensional materials were the most common, such as flexible electronic system designed by Yuxiang Shi et al., which could be worn on the arm for virtual environment control. Additionally, we highlighted waterproof textiles designed by Feng Wen et al., and the flexible control patch designed by Qiongfeng Shi et al., which could be worn on the human body to realize energy collection. The advantages of these designs included their small size, wearability, flexible operation, and low cost. These designs mainly differed in their wearable portions, as some were gloves and some were two-dimensional patches worn on the arm. In addition, the application scenarios were different, as some were used to control the computer screen, some were used for energy harvesting in real sports, and others were used in the field of biomedicine to realize simulated surgery.

## 6. Future Applications for TENG

**Figure 12:**
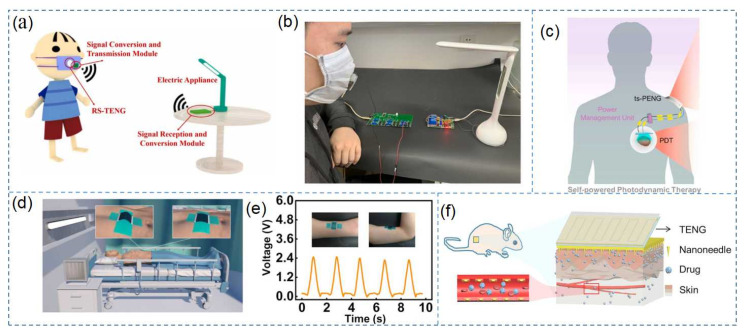
Use of TENG in future medical applications. (**a**,**b**) TENG-based respiratory monitoring face mask. Reprinted with permission from ref. [132]. Copyright 2022 Nano Energy; (**c**) TENG-based cancer treatment device. Reprinted with permission from ref. [133]. Copyright 2022 Nano Energy; (**d**) TENG-based bandage sensor; (**e**) bandage sensor attached to the arm to measure the voltage signal. Reprinted with permission from ref. [134]. Copyright 2022 Nano Energy; (**f**) schematic diagram of drug delivery based on TENG.

TENG may likely be widely used in the medical field in the future. In recent years, the novel coronavirus epidemic has swept the world, which has significantly impacted human health. To monitor the health status of patients, TENG-based breathing sensors could be integrated with masks to monitor patients in real time. The respiratory status of a patient is shown in Figure 12a,b [132]. Integrating TENG with cancer treatment equipment could also be used to monitor their condition at home for an extended period without the need for an external power supply, as shown in Figure 12c [133]. A TENG-based biodegradable bandage sensor could be used to monitor the physiological state of the human body in real time, and the sensor could be reused, as shown in Figure 12d,e [134]. The TENG-based electroporation system has been shown to deliver drugs to mice, as presented in Figure 12f. If TENG could be combined with AR and VR in telemedicine and surgical treatment applications, it would benefit more patients. Therefore, TENG experiments in the medical field may likely be implemented into practical applications, offering significant contributions to the medical field and a benefit to human health.

## 7. Summary and Outlook

This paper described in detail the types of materials that can be used for triboelectric nanogenerators, including PTFE and PDMS, which are the most commonly used triboelectric materials for the triboelectric layer in TENGs, especially for TENGs operating in transverse sliding and independent triboelectric layer modes. In addition to material selection, a better understanding of materials is needed to improve the triboelectrification effect, such as chemical etching and coating, as it can be used to fit the two friction materials more closely together to improve the TENG power output. This paper also described the applications of triboelectric nanogenerators in AR, VR, and other wearable electronic devices, and summarized the design methods of TENGs as self-powered sensor modules in these devices.

The current method of designing a self-powered sensor module is relatively complicated and difficult to integrate with electronic equipment; therefore, it is difficult to realize for large-scale industrial production. The design and integration of triboelectric nanogenerators requires further research. Moreover, the stability and reliability of TENGs may seriously degrade due to the unavoidable mechanical and environmental effects that the devices are often subjected to during use. These factors can easily result in material and device failure, causing problems such as lower output voltage, shortened lifespan, and potential safety hazards. Therefore, TENG robustness and reliability are issues that must still be addressed. In the future, nanogenerators may require more in-depth research regarding applications in the field of wearable electronic equipment, especially in the field of medical detection devices. The pursuit of future medical detection devices is focused on lightweight and family-oriented applications, which is not sufficient for every patient. Users want to check their own health status; thus, if wearable nanogenerators are to have better medical field applications in the future, they must be inseparable from the choice of materials, such as those with good biocompatibility, good sweat resistance, flexibility, and mechanical stability. At the same time, the corrosion resistance and degradation of materials in physiological environments should be considered, and new device integration and packaging technologies should be developed to fully realize the wide use of TENGs.

## Data Availability

No new data were created or analyzed in this study. Data sharing is not applicable to this article.
